# The health economic potential of harnessing placebos in treatment of ADHD

**DOI:** 10.1192/j.eurpsy.2021.1281

**Published:** 2021-08-13

**Authors:** J. Hamberger, K. Meissner, T. Hinterberger, T. Loew

**Affiliations:** 1 Research Section Of Applied Consciousness Sciences, Department Of Psychosomatic Medicine, University Clinic Regensburg, Regensburg, Germany; 2 Division Of Health Promotion, University of Applied Sciences Coburg, Coburg, Germany; 3 Institute Of Medical Psychology, Ludwig-Maximilians-University Munich, München, Germany; 4 Department Of Psychosomatic Medicine And Psychotherapy, University Clinic Regensburg, Regensburg, Germany

**Keywords:** Placebo effect, health economics, ADHD

## Abstract

**Introduction:**

Placebo research investigated the underlying mechanisms of placebo effects, but they are rarely used to optimize treatments. Ethical and legal concerns have been raised, but research demonstrated that placebo mechanisms can be used without patients’ deception: Experimental studies showed that half of drugs in treatment of attention-deficit/hyperactivity disorder (ADHD) combined with open-label placebos could be as effective as standard medication to reduce ADHD symptoms.

**Objectives:**

To estimate the health economic advantages of harnessing the combination of open-label placebos with standard medication in ADHD.

**Methods:**

For preliminary estimation of the mean treatment costs, the 12-months prevalence of ADHD in children and adolescents aged 5 to 14 years as well as the percentage of medication treatments were extracted from the literature. Mean treatment costs per patient and year were calculated for four treatment plans (different drugs and dosages) with both treatment with standard medication and half of drugs in combination with placebos.

**Results:**

A 12-months prevalence of 4.3% equals around 260,000 children and adolescents with a compulsory health insurance in Germany. Of those, around 40-50% are equally treated with two standard drugs and two different dosages. Full standard drug treatments cost around 119 million EUR, and treatment with half of drugs in combination with placebos cost around 66 million EUR.

**Conclusions:**

The combination of open-label placebos with half of standard medication could considerably reduce health costs. Reduction of side effects still must be considered. However, current studies are of experimental nature and lasted for no longer than two weeks.

